# Personalia

**Published:** 1914-06-06

**Authors:** 


### Personalia.

MR. J. A. PEEL, vice-chairman of the Tolworth Joint Hospital Board, has been elected chairman for the year.

DR. HENRY R. HUTTON, who has been a member of the board of governors and honorary physician to the Manchester Children's Hospital for the last thirty years, has been appointed honorary consulting physician upon his retirement from the visiting staff.

AT a meeting of the election committee of the General Hospital, Birmingham, held recently, when Mr. J. B. Clarke, vice-president, was in the chair, Dr. A. Stanley Barnes, M.D., F.R.C.P., senior assistant physician, was elected hononary physician for a term of fifteen years in place of Sir Robert M. Simon, M.D., resigned. Dr. Stanley Barnes was appointed assistant physician to the hospital in June 1906. He is honorary curator of the Pathological Museum of the University, a fellow of the Royal Society of Medicine, and a member of the British Medical Association. He formerly held the posts of demonstrator in physiology at Mason's College, resident medical officer at the National Hospital for the Paralysed and Epileptic, London, and pathologist at the Queen's Hospital, Birmingham.

### MEMORIAL TO A DOCTOR.

WITH the object of discussing the means of commemorating the work of the late Dr. Percy Warner, of Woodford, a meeting was recently held at the Wilfred Lawson Hotel, when it was decided that the memorial should take the form of a fund, to be known as the "Percy Warner Samaritan Fund," to be controlled by the authorities of the Woodford Jubilee Hospital. It was also decided that a portion of the moneys subscribed should be used to erect a tablet to the late Dr. Warner within the hospital.

